# Efficient Removal of Heavy Metals from Aqueous Solution Using Licorice Residue-Based Hydrogel Adsorbent

**DOI:** 10.3390/gels9070559

**Published:** 2023-07-09

**Authors:** Xiaochun Yin, Ting Ke, Hai Zhu, Pei Xu, Huiyao Wang

**Affiliations:** 1 Department of Civil Engineering, New Mexico State University, Las Cruces, NM 88003, USA; pxu@nmsu.edu; 2 School of Public Health, Gansu University of Chinese Medicine, Lanzhou 730000, China; k19060388@126.com (T.K.); zhuhai19960429@163.com (H.Z.)

**Keywords:** hydrogel, Chinese herb, adsorbent, heavy metals, cellulose, remove

## Abstract

The removal of heavy metals through adsorption represents a highly promising method. This study focuses on the utilization of an abundant cellulose-rich solid waste, licorice residue (LR), as a natural material for hydrogel synthesis. To this end, LR-EPI hydrogels, namely, LR-EPI-5, LR-EPI-6 and LR-EPI-8, were developed by crosslinking LR with epichlorohydrin (EPI), specifically targeting the removal of Pb, Cu, and Cr from aqueous solutions. Thorough characterizations employing Fourier-transform infrared spectroscopy (FTIR) and scanning electron microscopy confirmed the successful crosslinking of LR-EPIs by EPI, resulting in the formation of porous and loosely structured hydrogels. Batch studies demonstrated the high efficacy of LR-EPI hydrogels in removing the three heavy metal ions from aqueous solutions. Notably, LR-EPI-8 exhibited the highest adsorption capacity, with maximum capacities of 591.8 mg/g, 458.3 mg/g, and 121.4 mg/g for Pb^2+^, Cr^3+^, and Cu^2+^, respectively. The adsorption processes for Pb^2+^ and Cu^2+^ were well described by pseudo-second-order kinetics and the Langmuir model. The adsorption mechanism of LR-EPI-8 onto heavy metal ions was found to involve a combination of ion-exchange and electrostatic interactions, as inferred from the results obtained through X-ray photoelectron spectroscopy and FTIR. This research establishes LR-EPI-8 as a promising adsorbent for the effective removal of heavy metal ions from aqueous solutions, offering an eco-friendly approach for heavy metal removal and providing an environmentally sustainable method for the reutilization of Chinese herb residues. It contributes to the goal of “from waste, treats waste” while also addressing the broader need for heavy metal remediation.

## 1. Introduction

Heavy metals, such as Pb, Cu, and Cr, are generated through industrial activities, leading to significant environmental contamination and adverse effects on aquatic ecosystems. These metals pose a serious threat due to their non-biodegradable nature and ability to accumulate in living organisms, even at trace concentrations or levels below detection limits [1,2,3,4]. The presence of these metals can disrupt organism functions and result in toxicological effects [5]. Consequently, the development of cost-effective and efficient technologies for the removal of dissolved heavy metals is of utmost importance [6].

Adsorption is a widely used technique for extracting heavy metals from various water resources and wastewater due to its advantages such as short retention time, economic feasibility, high removal efficiency, and potential for metal recovery [7,8,9,10,11]. There has been considerable interest in the development of low-cost adsorbents derived from plants or agricultural solid wastes, primarily due to their wide availability, low market value, and potential for large-scale wastewater treatment [5]. Examples of such environmentally sustainable adsorbents include agricultural wastes like bagasse, potato peels, apple peels, and Chinese herb residues, which offer benefits such as cost-effectiveness, efficiency, biocompatibility, and biodegradability [12,13,14,15,16]. Moreover, their abundant functional groups (such as -OH, -COOH, and -NH_2_) contribute to their enhanced adsorption performance [17]. Unlike bagasse, potato peels, and apple peels, which often suffer from mechanical weakness and limited reusability, Chinese herb residues derived from the pharmaceutical industry’s extraction and decoction processes exhibit good chemical stability and mechanical strength [18]. The pharmaceutical industry generates approximately 30 million tons of Chinese herb residue annually, leading to a significant economic burden with regard to proper treatment and disposal [19]. Considering the increasing generation of Chinese herb residue, it is imperative to prioritize reuse due to economic and environmental constraints on landfilling [20,21,22]. Therefore, the recovery and reuse of Chinese herb residues for the production of useful products not only helps reduce environmental pollution but also promotes the sustainable management of waste materials.

Previous studies have explored the effectiveness of adsorbents based on Chinese herb residue in the removal of environmental pollutants [23,24,25,26,27]. Researchers have investigated the removal of copper and lead from water using chemically or biologically modified Chinese herb residue-based adsorbents [15]. Additionally, extracted cellulose from Chinese herb residue has been utilized in the preparation of adsorbents specifically targeting lead removal [28]. However, it has been observed that the adsorption capacity of simply modified Chinese herb residue is limited due to the presence of lignin and hemicellulose that encase the cellulose, as well as the by-products generated during the extraction process. Consequently, the development of high-performance adsorbents derived from Chinese herb residue has emerged as a pressing need to address this issue.

Various forms of adsorbents, including activated carbon, nanoparticles, and hydrogels, have been extensively studied for their efficacy in the removal of heavy metals [29,30,31]. However, compacted activated carbon adsorbents exhibited slow adsorption dynamics due to the limited diffusion of metal ions into small pores [32]. This extended equilibrium time for activated carbon was typically around 5 h [33]. Additionally, the high processing cost and challenges associated with regeneration contributed to the overall expense of drinking water treatments [34]. On the other hand, hydrogels with their three-dimensional network structures demonstrated high efficiency in removing heavy metals from aqueous solutions [35,36,37,38]. The presence of abundant functional groups such as -OH, -NH_2_, and-COOH in hydrogels promoted high permeability, allowing for the optimal exposure of internal adsorption sites to metal ions and ensuring the efficient removal of heavy metals from water [20]. Moreover, our previous study showcased the excellent performance of hydrogels in heavy metal removal using whole Chinese herb residue [39]. Hence, hydrogels represent a promising material for the effective removal of heavy metals from water.

However, due to the abundance of hydroxyl groups, the utilization of natural cellulose in the preparation of hydrogels based on Chinese herb residue requires chemical modification [40,41]. Chemical crosslinking enables cellulose to form a hydrogel with enhanced adsorption capacity and mechanical stability [42]. Commonly used crosslinking agents include citric acid, epichlorohydrin, and glutaraldehyde [43,44]. In a previous study, the crosslinking of cellulose using epichlorohydrin (EPI) had shown improvements in adsorption capacity, chemical stability, pore size distribution, and mechanical properties [45]. In comparison, glutaraldehyde raised concerns regarding the potential presence of toxic residues after the reaction [46]. Moreover, the use of citric acid or cellulose crosslinking required an elevated temperature to achieve higher crosslinking efficiency, which may lead to cellulose degradation [47]. Based on the aforementioned factors, EPI is considered a superior crosslinker for hydrogel preparation.

The objectives of this study are as follows: (i) to prepare the hydrogel adsorbents using solid waste licorice residue; (ii) to assess the removal performance of Pb^2+^, Cu^2+^, and Cr^3+^ by LR-EPI hydrogels through kinetics and sorption isotherms; (iii) to investigate the effects of pH, contact time, and initial concentration on the removal of Pb^2+^, Cu^2+^, and Cr^3+^ through static sorption experiments; and (iv) to discuss the adsorption mechanisms involved in the adsorption of heavy metals.

## 2. Results and Discussion

### 2.1. Structure and Chemistry Composition Analysis

**Figure 1:**
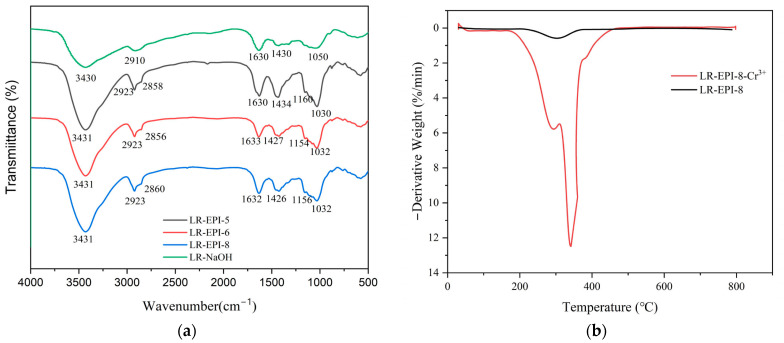
(**a**) FTIR spectra of LR-EPI-5, LR-EPI-6, LR-EPI-8, and LR-NaOH and (**b**) DTA of LR-EPI before and after adsorption (Cr^3+^).

The FTIR spectra of the prepared adsorbents exhibit bands around 3431 cm^−1^, which can be attributed to O-H stretching (Figure 1a). Furthermore, the bands at 1426, 1162, and 1032 cm^−1^ correspond to O-H bending, as well as symmetrical and asymmetrical C-O-C stretching vibrations. The presence of ether bonds resulting from EPI crosslinking is indicated by peaks around 1032 cm^−1^ in the crosslinked hydrogels (LR-EPI-5, LR-EPI-6, and LR-EPI-8), providing additional evidence for the occurrence of the crosslinking reaction compared to non-crosslinked cellulose (LR-NaOH). The peaks around 1030 cm^−1^ are associated with the stretching vibration of C-O-C, which forms as a result of the crosslinking between the -OH of LR and the ether bonds of EPI. Additionally, the chemical crosslinking of cellulose with ECH is demonstrated by the appearance of new bands at approximately 2923 and 2860 cm^−1^, which are assigned to the CH_2_ group [47]. The bands around 1630 cm^−1^ can be attributed to the stretching vibration of C=O from the ester groups in the cellulose. The bands detected at 2920 cm^−1^ are associated with C-H stretching and bending. The similar FTIR spectra of non-crosslinked LR and the hydrogels suggest that the fundamental structural units of cellulose are preserved.

Thermogravimetric analysis (TGA) was conducted to characterize the hydrogels. The weight loss behavior of the samples was observed as the temperature increased from 29 °C to 788℃, revealing three distinct stages: 29–240 °C, 240–500 °C, and 500–788 °C (Figure S4). The initial stage of weight loss can be attributed primarily to the evaporation of water contained within the samples or the removal of residual absorbed water. The weight loss observed in the range of 240–500 °C corresponds to the degradation of the cellulose molecular framework and EPI constituents. Subsequently, in the final stage, the hydrogel undergoes a gradual decomposition process. Upon adsorption of Cr^3+^, it was observed that LR-EPI-8 experienced greater weight loss during the third stage compared to its pre-adsorption state. Furthermore, analysis of the differential thermal analysis (DTA) curve (Figure 1b) also revealed three distinct stages: 29–240 °C, 290–480 °C, and 480–788 °C. During the first stage, the hydrogel exhibited stability. Peaks observed in the second stage can be attributed to the combustion of the hydrogel accompanied by the release of heat, potentially leading to the decomposition of the crosslinking structure of hydrogels, with LR-EPI-8-Cr^3+^ showing greater weight loss compared to LR-EPI-8. During the final stage, the hydrogel also exhibited stability. This high weight loss for LR-EPI-8-Cr^3+^ under thermal stability can be attributed to the immersion of the hydrogel in the solution for a duration of 2 h, resulting in the dissolution of oligomers from the hydrogel matrix. Consequently, the adsorption process induced a looser structural arrangement of the hydrogel compared to its original state, ultimately leading to a decrease in thermal stability.

**Figure 2:**
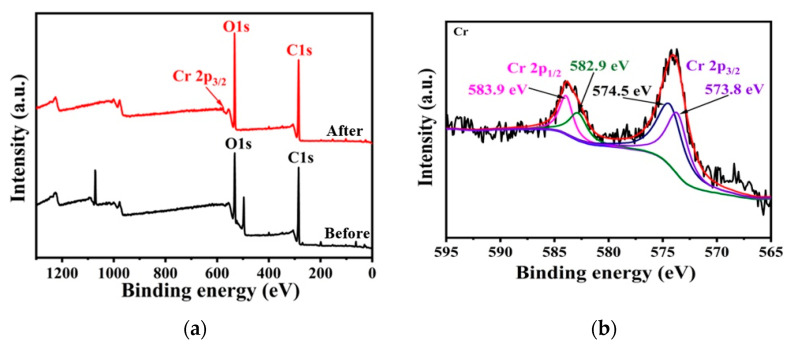

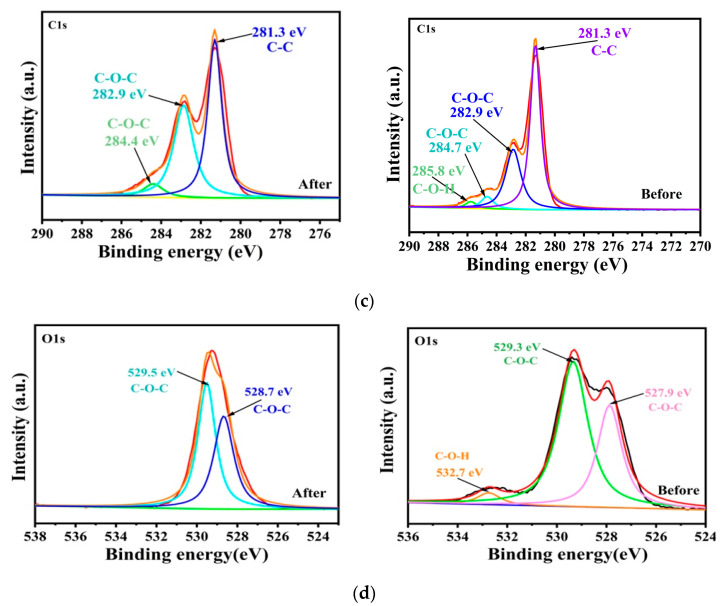
XPS spectra of (**a**) all elements of LR-EPI-8 before and after Cr^3+^ adsorption, (**b**) Cr 2p_1/2_ and Cr 2p_3/2_ for LR-EPI-8 after Cr^3+^ adsorption, (**c**) C 1s, and (**d**) O 1s for LR-EPI-8 before and after the adsorption.

X-ray photoelectron spectroscopy (XPS) analysis was utilized to gain insights into the interactive behaviors between the adsorbents and adsorbates. The survey spectrum (Figure 2a) revealed notable changes when comparing pristine LR-EPI-8 with LR-EPI-8-Cr. Specifically, a new peak attributed to Cr at 574.5 eV was observed in LR-EPI-8-Cr, indicating the successful adsorption of Cr^3+^ onto the surface of LR-EPI-8. In Figure 2b, the Cr 2p_1/2_ and Cr 2p_3/2_ XPS spectrum exhibited two distinct peaks at binding energies of 582.9, 583.9, 574.5, and 573.8 eV, which were assigned to Cr 2p_1/2_ and Cr 2p_3/2_, respectively. The C 1s XPS peaks of LR-EPI-8 could be deconvoluted into four peaks (i.e., 281.3 eV (C-C), 282.9 eV (C-O-C), 284.7 eV (C-O-C), and 285.8 eV (C-O-H)) (Figure 2c). It was evident that the characteristic peak of C-O-H disappeared after Cr^3+^ adsorption, while the 282.9 eV (C-O-C) peak remained unchanged, indicating the preservation of the cellulose structure. Additionally, the 532.7 eV (C-O-H) peak vanished, suggesting that the primary adsorption group for Cr^3+^ was the -OH group (Figure 2d).

### 2.2. Morphology and Structure of Hydrogels

**Figure 3:**
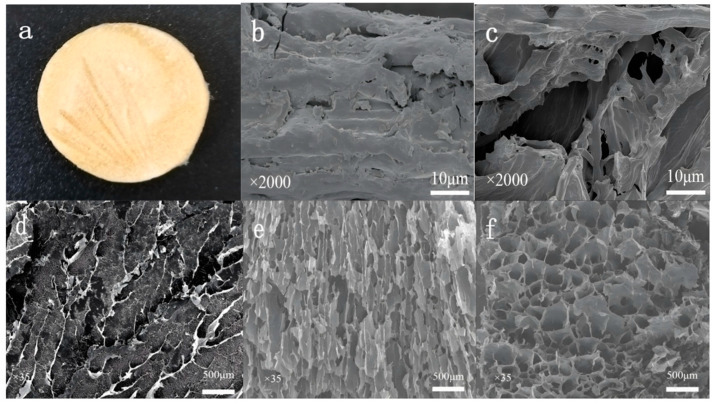
Photograph of (**a**) lyophilized LR-EPI-8; SEM images of (**b**) LR, (**c**) LR-NaOH, (**d**) LR-EPI-5, (**e**) LR-EPI-6, (**f**) LR-EPI-8.

The morphology of LR-EPI was captured in photographs shown in Figure S2a–f. As the concentration of LR increased, the crosslink density of hydrogels LR-EPI-6 and LR-EPI-8 also increased. The gel yields of the three hydrogels are approximately 88.6% (LR-EPI-5), 89.8% (LR-EPI-6), and 90.7% (LR-EPI-8). These results indicate that LR-EPI-8 had the highest crosslink density among the three hydrogels, as more cellulose was crosslinked with EPI, resulting in an increased crosslink density. This effectively prevented mechanical failure or complete dissolution of the adsorbent materials. After lyophilization (Figure 3a), the hydrogels lost water, resulting in a loose and porous three-dimensional network structure. This exposed more of the -OH group, which facilitated the removal of heavy metal ions.

The surface structures of LR, LR-NaOH, LR-EPI-5, LR-EPI-6, and LR-EPI-8 were characterized using SEM (Figure 3b–f). LR exhibited a relatively dense structure and low adsorption capacity. The structure of LR-NaOH was looser compared to LR, and there were a few pores on its surface, resulting from the modification-induced collapse of the lignocellulose structure [15]. All hydrogels (LR-EPI-5, LR-EPI-6, and LR-EPI-8) showed three-dimensional porous structures. Regardless of the EPI amount, the crosslinked hydrogels (LR-EPIs) had higher adsorption capacities compared to non-crosslinked cellulose (LR and LR-NaOH) [48]. Among the hydrogels, the pore size increased with increasing LR concentration, and LR-EPI-8 exhibited more homogeneous jelly-like and regular pores compared to LR-EPI-5 and LR-EPI-6. This feature provided LR-EPI-8 with more adsorption sites and exposed functional groups for its effective interaction with contaminants such as Pb^2+^, Cu^2+^, and Cr^3+^, resulting in a high adsorption capacity. Additionally, the surface chemical properties of the hydrogels could be influenced by the intermolecular bonds formed through crosslinking. When the cellulose/EPI ratios approached the stoichiometric proportion, the hydrogel exhibited a higher surface area and the best adsorption capacity [49]; therefore, the adsorption capacities of hydrogels were enhanced.

### 2.3. Adsorption Performance of LR-EPI Hydrogels

#### 2.3.1. Effect of Solution pH on Adsorption

**Figure 4:**
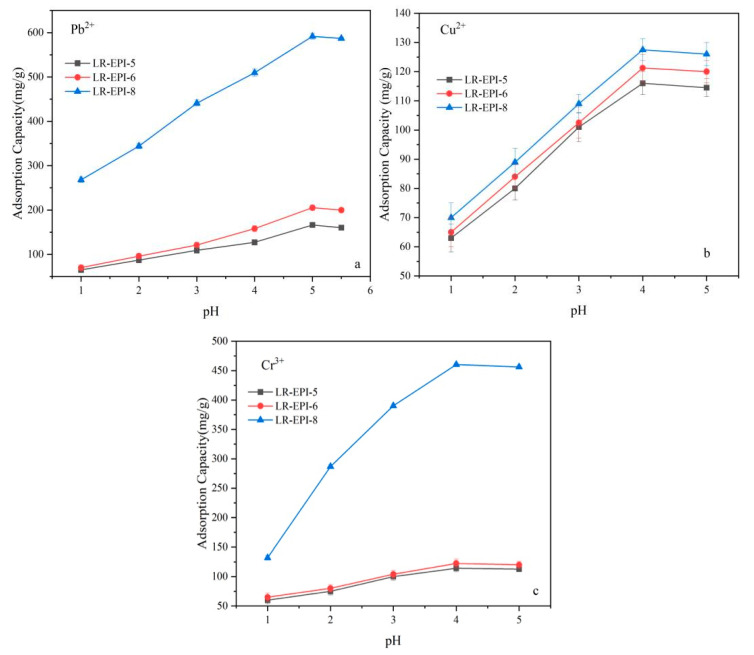
Effect of pH on (**a**) Pb^2+^, (**b**) Cu^2+^, and (**c**) Cr^3+^ adsorption. Adsorption conditions: 300 mg/L of Pb^2+^ solution and 700 mg/L of Cu^2+^ and Cr^3+^; temperature, 25 °C; shaking time, 60 min; adsorbents, 0.01 g; contact time, 2 h; volume of adsorbate, 25 mL.

The removal of contaminants from aqueous solution through adsorption is greatly influenced by the solution pH, which impacts the surface charge of the hydrogels, the degree of ionization, and the speciation of the adsorbate. Figure 4 depicted the adsorption capacities of LR-EPI-5, LR-EPI-6, and LR-EPI-8 for Pb^2+^, Cu^2+^, and Cr^3+^ as a function of the pH ranging from 1.0 to 5.0.

The adsorption capacities of LR-EPI-8 were higher compared to LR-EPI-5 and LR-EPI-6. As the pH increased from 1.0 to 5.0, the adsorption capacities of the hydrogels for Pb^2+^, Cu^2+^, and Cr^3+^ also increased. Among the three adsorbents, LR-EPI-8 exhibited the highest adsorption capacities for all three heavy metal ions. Specifically, the adsorption capacities for Pb^2+^, Cu^2+^, and Cr^3+^ increased from 268.1 mg/g, 70.4 mg/g, and 132.0 mg/g to 591.8 mg/g, 127.5 mg/g, and 460.3 mg/g, respectively. On the other hand, LR-EPI-5 demonstrated the lowest adsorption capacities with values increasing from 65.2 mg/g, 63.5 mg/g, and 60.3 mg/g to 166.3 mg/g, 116.2 mg/g, and 114.3 mg/g for Pb^2+^, Cu^2+^, and Cr^3+^, respectively.

This phenomenon can be explained by the alteration in the surface active sites of the hydrogels. The primary active sites are -OH groups, which, at lower pH values, become protonated by H^+^ ions, resulting in a positively charged surface of the adsorbent. Consequently, the number accessible for heavy metal ion uptake decreases. Moreover, the abundance of H^+^ ions can compete with metal ions through ion-exchange reactions. As depicted in Figure S3, LR-EPI -8 exhibited a negative zeta potential within a pH range of 2.0–5.0. As the pH increased, the negatively charged active sites increased, enhancing the electrostatic attraction between the negatively charged hydrogel surface and the positively charged contaminants [50]. However, at higher pH values (above 4.0 or 5.0), excess anions (OH^−^) covered the binding sites of the hydrogel, reducing the removal of contaminants due to electrostatic repulsion [51]. The sedimentation of heavy metals will occur when the pH exceeds 5.5; therefore, to ensure the accuracy of the results, it was deemed necessary to limit the pH range within this boundary. For the subsequent experiments, a pH of 4.0 was selected as the optimal pH for Cu^2+^ and Cr^3+^ solutions.

Among the three hydrogels, LR-EPI-8 exhibited the highest adsorption capacity for heavy metals, with the removal of Pb^2+^ being higher than that of Cu^2+^ and Cr^3+^. One reason for this is that increasing the LR concentration resulted in an increase in active groups and surface areas of hydrogels [52], as well as an enhanced swelling ratio (LR-EPI-8: 27.27, LR-EPI-5: 19.60, and LR-EPI-6: 24.28), which can be attributed to the chemical crosslinking that lengthens the distance between cellulose chains [53]. This ultimately led to a higher adsorption performance of LR-EPI-8 compared to LR-EPI-5 and LR-EPI-6. Another reason is that the properties of heavy metal ions, including electronegativity, ionization potential, and ionic radius [54], caused the hydrogels to exhibit higher selectivity for the removal of Pb^2+^ compared to Cu^2+^ and Cr^3+^.

To mitigate the occurrence of heavy metal sedimentation beyond a pH of 5.5, it was deemed essential to confine the pH range within this threshold. Consequently, for subsequent experiments, a pH value of 4.0 was identified as the optimal condition for Cu^2+^ and Cr^3+^ solutions, while a pH of 5.0 was determined to be the optimal condition for Pb^2+^ solutions.

#### 2.3.2. Effect of Contact Time on Adsorption

**Figure 5:**
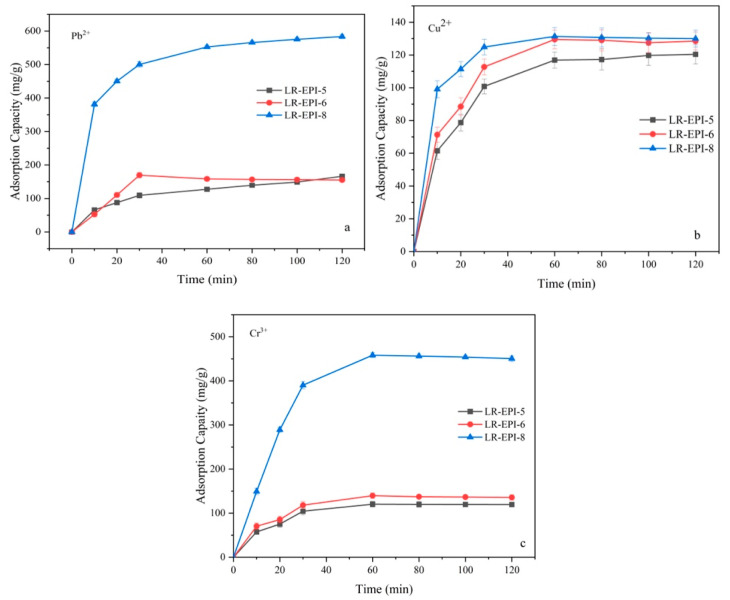
Effect of contact time on (**a**) Pb^2+^, (**b**) Cu^2+^, and (**c**) Cr^3+^ adsorption. Adsorption conditions: 300 mg/L of Pb^2+^, 700 mg/L of Cu^2+^ and Cr^3+^; temperature, 25 °C; shaking time, 60 min; adsorbent, 0.01 g; volume of adsorbate, 25 mL.

Contact time is another significant factor that influences the adsorption equilibrium. The adsorption of Pb^2+^, Cu^2+^, and Cr^3+^ was conducted for a duration of 120 min. The adsorption capacities of the LR-EPI hydrogels increased steadily with the duration of adsorption until reaching equilibrium, as shown in Figure 5. As the binding sites became nearly saturated pollutants, the active sites of the hydrogels reached an increasing saturation, resulting in the adsorption capacities reaching equilibrium. With a longer contact time, more heavy metal ions were bound to the surface of the hydrogels, thereby increasing the adsorption capacities. Additionally, during the adsorption process, swelling and adsorption might occur simultaneously. As the swelling ratio increased, the heavy metal ions gradually entered gel pores with water [40]. The equilibrium adsorption time for Pb^2+^, Cu^2+^, and Cr^3+^ on LR-EPI-5 and LR-EPI-6 was approximately 60 min due to their lower -OH content compared to LR-EPI-8. In comparison to LR-EPI-5 and LR-EPI-6, LR-EPI-8 exhibited a faster adsorption rate (120 min for Pb^2+^ and 60 min for Cu^2+^ and Cr^3+^) and much higher maximum adsorption capacities (583.6 mg/g and 458.3 mg/g) for Pb^2+^ and Cr^3+^. The possible reason for this is that the active groups of LR-EPI-8 increased with the LR concentration, and Pb^2+^ and Cr^3+^ were more selectively adsorbed by the hydrogels.

#### 2.3.3. Adsorption Kinetics

To analyze the experimental data and study the adsorption rate of the LR-EPI hydrogels, dynamic models were applied. Among the various kinetic models used for solid–liquid interactions, the pseudo-first-order (Equation (1)) and pseudo-second-order (Equation (2)) models are commonly utilized [55,56]. The pseudo-first-order dynamic model assumes that the adsorption rate is primarily governed by diffusion and mass transfer, while the pseudo-second-order assumes that chemical adsorption is the rate-controlling step.

(1) $$log(q_{e}-q_{t})=logq_{e}-\frac{k_{1}}{2.303}t$$

(2) $$\frac{t}{q_{t}}=\frac{1}{k_{2}{q_{e}}^{2}}+\frac{t}{q_{t}}$$

where *q_e_* (mg/g) is the adsorption amount at equilibrium, *q_t_* (mg/g) is the adsorption amount of a specific time *t*, and *k*_1_ (min^−1^) and *k*_2_ (g (mg-min)^−1^) are the pseudo-first-order and pseudo-second-order rate constant, respectively.

**Figure 6:**
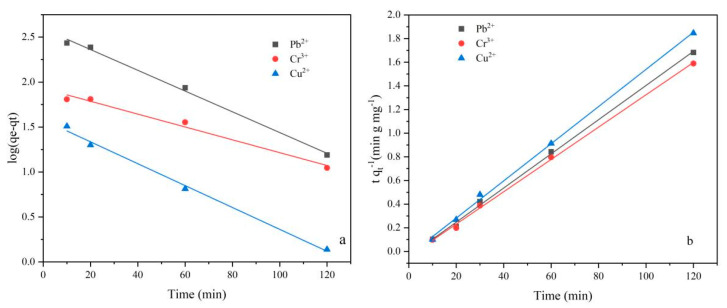
(**a**) Pseudo-first-order and (**b**) pseudo-second-order kinetic model for adsorption of heavy metals.

The fitting curves for the pseudo-first-order and pseudo-second-order kinetics are presented in Figure 6 and the parameters are shown in Table S1 (in supplementary materials), illustrating the adsorption of hydrogels for heavy metal ions. The pseudo-second-order dynamics equation exhibited a better fit, indicating chemical adsorption. Moreover, the pseudo-second-order kinetics suggested that the rate of chemical adsorption was proportional to the number of unoccupied active sites.

As depicted in Figures S5–S7 and Tables S2–S4, the adsorption processes of LR-EPIs can be divided into two stages for the three heavy metals. In the first stage, metal ions diffused onto the surface of the adsorbent. The diffusion model lines within the particles do not intersect with the origin, implying that the adsorption process is governed by both external and internal diffusion mechanisms. The overall adsorption process can be divided into two stages: initially, when the heavy metal ions attach to the external surface sites of the particles, the adsorption rate is relatively rapid; subsequently, as the adsorption on the outer surface reaches saturation, a gradual diffusion into the particle interior takes place. In this stage, the adsorption rate correlates with the boundary thickness (C), wherein an increase in C results in a gradual attenuation of the adsorption rate with the rate constant (k) approaching zero, eventually establishing a state of dynamic equilibrium. The model calculations consistently yield positive values for C, indicating that the rate-controlling steps of metal ion adsorption are determined by multiple stages, and particle internal diffusion is not the sole decisive factor.

#### 2.3.4. Effect of Initial Ion Concentration on Adsorption

**Figure 7:**
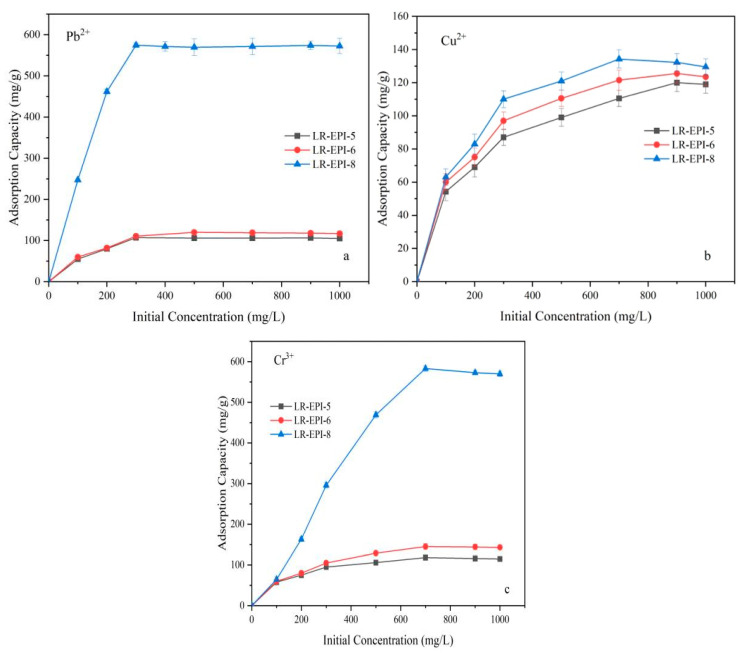
Effect of initial concentration on (**a**) Pb^2+^, (**b**) Cu^2+^, and (**c**) Cr^3+^ adsorption. Adsorption conditions: temperature, 25 °C; shaking time, 60 min; adsorbent, 0.01 g, volume of adsorbate, 25 mL.

The impact of the initial concentration of Pb^2+^, Cu^2+^, and Cr^3+^ on the adsorption was analyzed, with concentrations ranging from 100 to 1000 mg/L (Figure 7). It was observed that when the initial concentrations of Pb^2+^, Cu^2+^, and Cr^3+^ were 300 mg/L, 700 mg/L, and 700 mg/L, respectively, the adsorption processes reached equilibrium, indicating that the active sites were almost fully occupied. The highest adsorption capacities were achieved by LR-EPI-8 and LR-EPI-6, with values of 574.5 mg/g and 120.6 mg/g for Pb^2+^, 134.3 mg/g and 125.5 mg/g for Cu^2+^, and 583.5 mg/g and 145.8 mg/g for Cr^3+^, respectively. LR-EPI-5 exhibited a maximum adsorption capacity of 120.2 mg/g for Cu^2+^. Notably, LR-EPI-8 exhibited significantly higher adsorption capacities for Pb^2+^, Cr^3+^, and Cu^2+^ compared to LR-EPI-5 and LR-EPI-6, indicating a greater number of active sites available for metal ion removal and thereby resulting in its superior capacity. This phenomenon can be attributed to the presence of more positively charged ions which create a stronger driving force for the effective collision between ions and surficial functional groups of the hydrogel. Consequently, this promotes ions transfer between the two phases and collision between the metal ions and surficial functional groups of the hydrogels [40]. Once the active sites were fully occupied, the adsorption performance of the three hydrogels for heavy metals diminished [57], reaching equilibrium.

#### 2.3.5. Adsorption Isotherm

The adsorption isotherm is utilized to describe interface adsorption, which represents a physicochemical adsorption phenomenon resulting from the interaction between metal ions and hydrogel adsorbents. In this study, both the Langmuir and Freundlich isotherm models were examined. The Langmuir model [58] was applied to analyze single-layer adsorption systems, suggesting the presence of limited quantities of separated active sites without chemical interaction. On the other hand, the Freundlich model [59] accounted for the non-uniform surface during adsorption, making it suitable for describing multi-layer adsorption or a high-suction concentration system. The equations for the Langmuir (3) and Freundlich models (4) are as follows:

(3) $$\frac{C_{e}}{q_{e}}=\frac{1}{K_{L}q_{m}}+\frac{C_{e}}{q_{m}}$$

(4) $$ln\;q_{e}=ln\;K_{F}+\frac{1}{n}ln{\;C}_{e}$$

where *q_e_* (mg/g) is the adsorption amount at equilibrium, *C_e_* (mg/L) is the concentration at equilibrium, *K_L_* is the Langmuir constant, *q_m_* (mg/g) is the maximum adsorption amount covering the entire surface, *R_L_* is a separation coefficient or an equilibrium parameter, *C*_0_ (mg/L) is the initial concentration of heavy metal ions, and *K_F_* and *n* are Freundlich constants.

**Figure 8:**
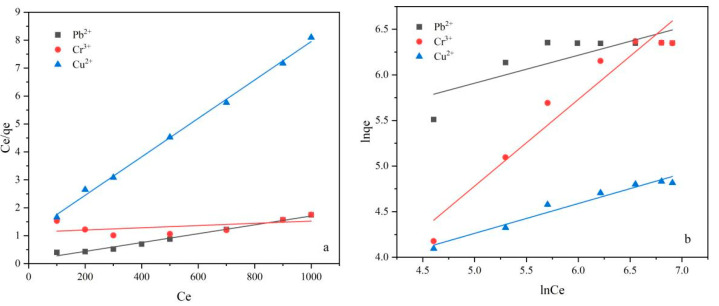
(**a**) Langmuir and (**b**) Freundlich isotherm models of heavy metal ion adsorption on LR-EPI hydrogels.

Figure 8 and Table S5 present the fitting results of the two models. Based on the correlation coefficient (*R^2^*), the Langmuir model provided the optimal fit for describing the adsorption process for Pb^2+^ and Cu^2+^, indicating single-layer adsorption. Moreover, both *R_L_* values being less than 1 and *n* being greater than 1 indicated favorable adsorption characteristics of the adsorbents. However, for Cr^3+^, although the adsorption process was also single-layer adsorption, it fit better with the Freundlich model. This observation could be attributed to excessive swelling of the hydrogel at low concentrations, leading to a significant measurement error. Furthermore, non-linear models were also applied to simulate the adsorption results, as shown in Figure S8 and Table S7. However, the obtained *R^2^* was too small, and the model evaluation parameters, such as the root-mean-square error, were too large. Hence, non-linear models are not suitable for accurately depicting the adsorption behavior of Cr^3+^. Additionally, the homogenous distribution of surface groups on the prepared hydrogels, as observed through FTIR and SEM analyses, supports the conclusion that the adsorption of Cr^3+^ is still monolayer adsorption, which is consistent with the findings of other studies [60,61].

In Table S6, the parameter *ΔG*° of LREPI-8 was determined for Pb^2+^ and Cu^2+^, resulting in values of −10.78 and −9.02 kJ/mol, respectively. Furthermore, the *ΔH*° values were calculated to be 1.52 kJ/mol for Pb^2+^ and 1.21 kJ/mol for Cu^2+^. The observed negative *ΔG*° and positive *ΔH*° values indicate that the adsorption of Pb^2+^ and Cu^2+^ on LR-EPI-8 is an endothermic process. This suggests that the adsorption is thermodynamically favorable and can be enhanced by increasing the reaction temperature. Additionally, the positive *ΔS*° values were determined to be 41.27 J/mol-K for Pb^2+^ and 34.33 J/mol-K for Cu^2+^. These findings suggest an increase in the disorderliness of the solid–liquid system, which can likely be attributed to the loss of hydration water associated with metal ions [23].

#### 2.3.6. The Effects of Salt Ions on the Adsorption Capacity of LR-EPI-8

**Figure 9:**
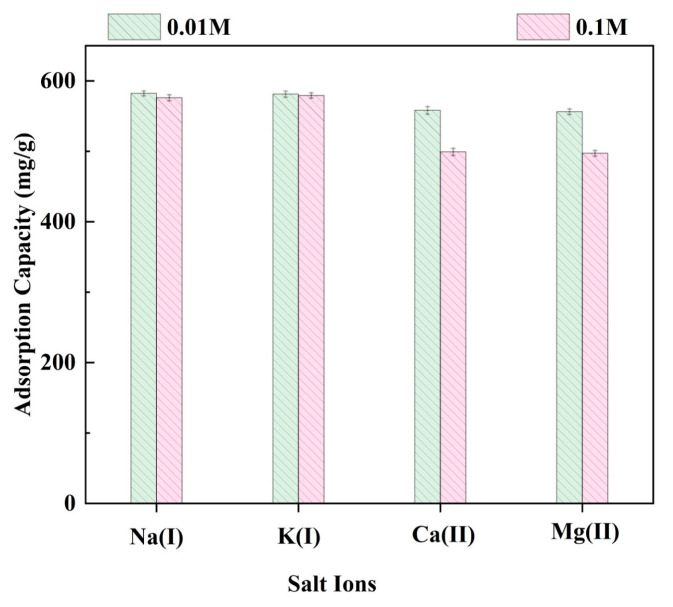
Effect of ionic species on adsorption capacity for Pb^2+^ using LR-EPI-8 (adsorption conditions: initial concentration of Pb^2+^, 300 mg/L; temperature, 25 °C; shaking time, 60 min; adsorbent, 0.01 g; pH = 5.0).

The interference capability of LR-EPI-8 against competing ions was investigated in the presence of various complex water matrices for the purpose of Pb^2+^ removal. To evaluate the competition between Pb^2+^ and other cations, such as Na^+^, K^+^, Ca^2+^, and Mg^2+^, salt ions were used at concentrations of 0.01 and 0.1 mol/L. As shown in Figure 9, LR-EPI-8 exhibited consistent Pb^2+^ adsorption capacities ranging from 98.3% to 94.1% in the presence of 0.01 mol/L salt ions, and these capacities decreased by 3% to 17% as the concentrations of interfering ions increased to 0.1 mol/L. These findings demonstrate that LR-EPI-8 possesses high selectivity and affinity for Pb^2+^, making it highly promising for the treatment of Pb-contaminated water.

#### 2.3.7. Regeneration

**Figure 10:**
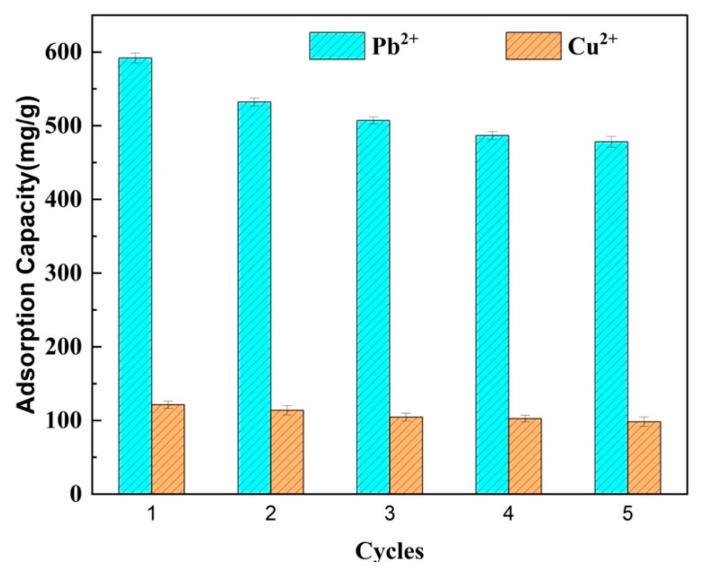
Regeneration of LR-EPI-8 for Pb^2+^ and Cu^2+^ adsorption.

The evaluation of regeneration is a crucial aspect when assessing the performance of adsorbents. In this study, the regeneration of LR-EPI-8 for Pb^2+^ and Cu^2+^ ions was examined through a series of five adsorption–desorption cycles. The initial adsorption capacity observed in the first cycle was considered to be 100%. Subsequently, the ratio of the adsorption capacity in each cycle to that of the initial cycle was calculated to assess the regeneration of the hydrogel. As depicted in Figure 10, the adsorption capacities exhibited a gradual decrease over successive cycles. However, even after undergoing five cycles of adsorption, the adsorption capacities for both Pb^2+^ and Cu^2+^ ions remained above 75%. This observation indicates that the magnetic hydrogels not only facilitated easy recovery, but also demonstrated excellent regeneration.

Table 1 presents a comparison between this study and other reported adsorbents in the literature, highlighting that the hydrogels synthesized in this study exhibited higher adsorption capacities for Pb^2+^, Cr^3+^, and Cu^2+^ compared to other hydrogel adsorbents. This finding underscores the potential of the LR-based hydrogel (LR-EPI-8) synthesized in this study as a promising adsorbent for the removal of heavy metals from aqueous solutions due to its remarkable adsorption capacity and rapid adsorption rate.

LR-EPI-8, a homogeneous hydrogel derived from natural material waste in a pharmacy, outperformed other adsorbents that involve the use of multiple chemicals for preparation. By employing only EPI as a crosslinking agent, LR-EPI-8 minimized chemical usage while achieving higher adsorption capacities for the three heavy metal ions. Furthermore, LR-EPI-8 utilizes the entire LR to prepare the hydrogel, thereby enhancing the reutilization of Chinese herb residue. Consequently, LR-EPI-8 based on LR emerges as an environmentally friendly and excellent adsorbent for the efficient removal of contaminants from water.

### 2.4. Adsorption Mechanism of LR-EPI Hydrogels

**Figure 11:**
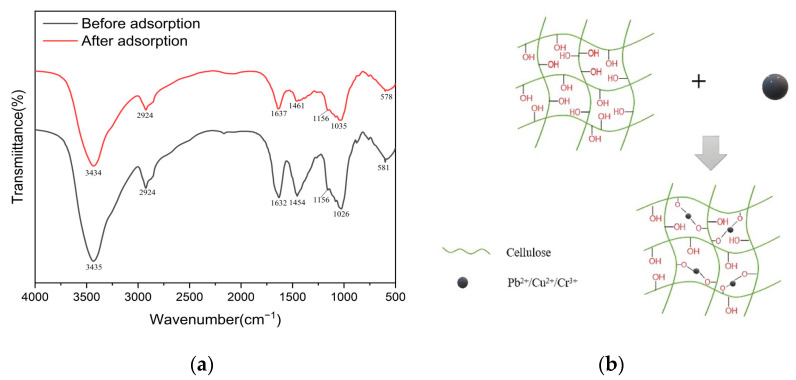
(**a**) FTIR spectra of LR-EPI-8 before and after adsorption of Cr^3+^ and (**b**) adsorption mechanism of LR-EPI hydrogels.

A thorough understanding of the adsorption mechanism of LR-EPI hydrogels for heavy metal ions is crucial for evaluating their adsorption performance. Generally, various interactions such as ion exchange, hydrophobic interactions, coordination interactions, and electrostatic interactions can occur during the adsorption process of LR-EPI hydrogels. In this case, the primary adsorption mechanism for LR-EPI hydrogels is believed to be the electrostatic interaction with heavy metal ions. This interaction occurs between specific functional groups in the monomer units of the adsorbents, resulting in the absorption or desorption of heavy metals through cation–anionic interactions [70]. By analyzing the FTIR spectra of LR-EPI-8 before and after the adsorption (Figure 11a), changes in the peak positions can be observed. The peaks at 1454 and 1026 cm^−1^, corresponding to O-H bending and symmetrical/asymmetrical C-O-C stretching vibrations, underwent alterations after adsorption. Additionally, the peak at 1632 cm^−1^, attributed to C=O from ester groups in cellulose, also experienced changes. From the change of peaks before and after adsorption, it is known that the major adsorption sites are -OH groups. Furthermore, as depicted in Figure S3, the LR-EPI-8 hydrogel exhibited a negative charge within the pH range of 2.0–5.0. With an increase in pH, the surface functional groups, such as -OH groups, lose protons due to the higher concentrations of OH^−^ ions, leading to the formation of anions like -O- on the surface of the adsorbent. This electrostatically charged surface facilitates the adsorption of positively charged Pb^2+^, Cu^2+^, and Cr^3+^ ions onto the negatively charged hydrogel. According to XPS before and after the adsorption for Cr^3+^, and by considering the spectra of C1s and O1s, we hypothesized that both carbon (C) and oxygen (O) elements were involved in the adsorption process with strong interactions between the -OH groups and Cr^3+^. Consequently, the adsorption processes were driven by an ion-exchange reaction coupled with the coordination of -OH with metal ions, while other forces such as electrostatic and hydrogen bonding also played roles. The possible adsorption mechanism is illustrated in Figure 11b.

## 3. Conclusions

A series of LR-EPI hydrogels were synthesized using licorice residue and EPI as a crosslinking agent. The surface of the hydrogels exhibited a rough, irregular, and porous structure, as observed in the SEM images. The adsorption capacities of the hydrogels for Pb^2+^, Cu^2+^, and Cr^3+^ were influenced by contact time, solution pH, and initial concentration. The LR-EPI hydrogels demonstrated high adsorption capacity, with LR-EPI-8 exhibiting the best performance. The maximum adsorption capacities of LR-EPI-8 for Pb^2+^, Cu^2+^, and Cr^3+^ were determined as 591.75 mg/g, 583.5 mg/g, and 134.25 mg/g, respectively. The order of maximum adsorption capacities for heavy metal ions on LR-EPI-8 was Pb^2+^ > Cr^3+^ > Cu^2+^. The adsorption processes followed the pseudo-second-order kinetic model, Langmuir isotherm model (for Pb^2+^ and Cu^2+^), and Freundlich isotherm model (for Cr^3+^), indicating the dominance of chemical adsorption. Ion exchange and other factors such as electrostatic interactions were considered as the possible mechanism for the adsorption process. Based on these findings, the LR-EPI-8 hydrogel shows great potential as a promising adsorbent for the removal of heavy metals from aqueous solutions.

## 4. Materials and Methods

### 4.1. Materials

The licorice used in the experiment was from Lanzhou Huirentang Pharmacy (Lanzhou, China). Urea (CH_4_N_2_O) was purchased from Yantai Shuangshuang Chemical Co., LTD (Yantai, China). Sodium hydroxide (NaOH), epichlorohydrin (EPI), ethanol absolute (C_2_H_6_O), and cupric nitrate (Cu(NO_3_)_2_) were purchased from Tianjin Damao Chemical Reagent Co., LTD (Tianjin, China). Lead nitrate (Pb(NO_3_)_2_) was acquired from Tianjin Kermel Chemical Reagent Co., Ltd. (Tianjin, China). Chromic chloride (CrCl_3_·6H_2_O) was purchased from Shanghai Zhongqin Chemical Reagent Co., LTD (Shanghai, China). Hydrochloric acid (HCl) and nitric acid (HNO_3_) were purchased from Baiyin Chemical Co., LTD (Baiyin, China). All chemical reagents were of analytical grade.

### 4.2. Preparation of Hydrogels

#### 4.2.1. Pretreatment of LR

The purchased licorice was subjected to several treatments for preparation. Firstly, it was washed with deionized water 3 times (as shown in Figure S1a) to remove impurities and contaminants. Then, it underwent three cycles of boiling, each lasting for 30 min, to mimic the conventional use of Chinese herbs and eliminate the active ingredient. Subsequently, 100 g of the dried licorice residue (LR) was combined with 1000 mL of 1 mol/L NaOH solution and stirred for 8h at room temperature. The pretreated LR was thoroughly rinsed with deionized water multiple times to remove any residual NaOH. After the rinsing process, the LR was dried (as depicted in Figure S1b), which was followed by crushing and sieving through a 200-mesh sieve (Figure S1c). The resulting material obtained from these procedures was named LR-NaOH.

#### 4.2.2. Dissolution of LR-NaOH

A total of 5 g of LR-NaOH powder was introduced into 50 mL of NaOH/urea solution (1:2, *w*/*w*). The mixture was stirred using a magnetic stirrer at room temperature at a speed of 200 rpm for 2 h. Following this, the solution was subjected to a freezing process at −20 °C, which was followed by thawing with continuous stirring at 0 °C for 2 h. This freezing and thawing step was repeated twice. The resulting solution was then treated in a microwave oven at 800 Watt for about 20 min, resulting in the formation of LR-NaOH-5 transparent solutions. Similarly, 6 g and 8 g of LR-NaOH powder were separately added to NaOH/urea solutions to produce LR-NaOH-6 and LR-NaOH-8 transparent solutions, following the same process.

#### 4.2.3. Preparation of LR-EPI Hydrogels

**Figure 12:**
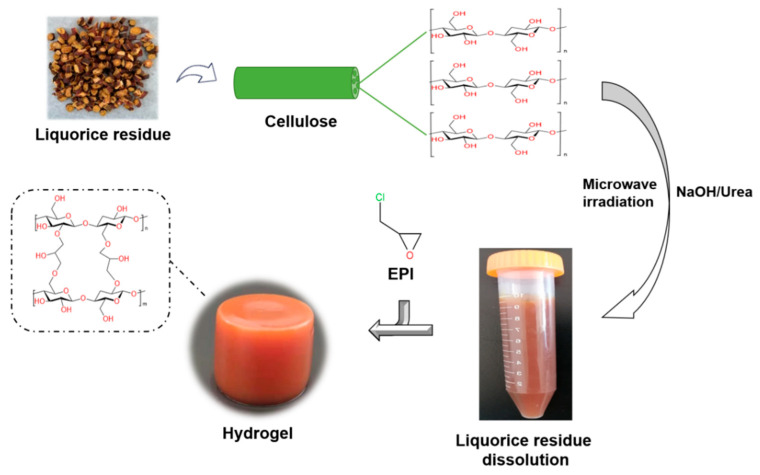
The synthesis scheme of LR-EPI hydrogels.

To prepare LR-EPI-5, 1.8 mL of EPI was added to LR-NaOH-5 at room temperature. The mixture solution was stirred for 30 min at a speed of 200 rpm. Subsequently, the beakers were sealed and placed in a water bath at 80 °C, allowing them to remain there for 6 h. Following this step, the samples were washed with ethanol to eliminate any residual chemicals and by-products. The hydrogels were then soaked in distilled water, with the water being changed twice. Finally, the hydrogels were subjected to lyophilization at −80 °C and 10 Pa for 24 h. The resulting dried samples were designated as LR-EPI-5. LR-EPI-6 and LR-EPI-8 were prepared using the same procedure. The synthesis scheme is depicted in Figure 12.

### 4.3. Characterization

The surface morphology and structure of the freeze-dried hydrogels were investigated using a scanning electron microscope (SEM, JSM-6701F, JEOL, Tokyo, Japan) operating at an accelerating voltage of 20 kV. To prepare the samples for analysis, samples were affixed to a copper sample holder using carbon tape and subsequently coated with a thin layer of gold. The structural properties of both non-crosslinked cellulose and hydrogels were assessed using Fourier-transform infrared spectroscopy (FTIR, Nicolet Nexus, Waltham, MA, USA). The lyophilized samples were pulverized into potassium bromide tablets, each containing 1% of the sample, and the resulting spectra were examined across a range of wavenumbers from 4000 cm^−1^ to 400 cm^−1^. The thermal stability of the samples was evaluated under a nitrogen atmosphere using PerkinElmer TGA-7 thermogravimetric instruments (PerkinElmer Cetus Instruments, Nor-walk, CT, USA) at a heating rate of 10 °C/min within the temperature range of 30–800 °C. Lastly, the surface chemistry of LR-EPI-8 before and after the adsorption of Cr^3+^ ions was analyzed using an X-ray photoelectron spectroscope (XPS, EscaLab 250 Xi+).

### 4.4. Equilibrium Degree of Swelling and Gel Fraction Yield of Hydrogels

The swelling properties of LR-EPI hydrogels were investigated by immersing approximately 0.05 g of the samples in 10 mL of deionized water for 24 h to reach equilibrium. The gravimetric method was employed to determine the equilibrium swellings. The lyophilized LR-EPIs were weighed as *W_d_*, while the hydrogels in their swollen state after reaching equilibrium were weighed as *W_s_*. The swelling ratio was calculated using Equation (1):

(5) $$S_{w}=\frac{W_{s}-W_{d}}{W_{d}}$$

where *S_w_* (%) is the swelling ratio, *W_s_* (g) is the weight of LR-EPI hydrogels after the swelling equilibrium was reached, and *W_d_* (g) is the weight of the lyophilized LR-EPIs.

The prepared LR-EPI hydrogels (0.05 g) were immersed in 10 mL of deionized water for 48 h and subsequently dried under vacuum at 40 °C until a constant mass was achieved. The dried hydrogels were then weighed. The gel fraction yield (GF) can be calculated as follows [71]:

(6) $$Gel\;fraction\;(\%)=\frac{mass\;of\;the\;dried\;sample\;after\;extration}{initial\;mass}\times 100$$

### 4.5. Zeta Potential Measurement

The zeta potential of LR-EPI-8 was determined using a SurPass Electrokinetic Analyzer (Anton Paar Trading Co., Ashland, VA, USA) to assess the charge distribution on the membrane surface at different pH levels. Prior to measurement, the hydrogel membrane was thoroughly rinsed with deionized water. The pH of the solution was adjusted by employing 0.1 M HCl and NaOH solutions. The desired pressure for the measurement procedure was set at 400 mbar.

### 4.6. Adsorption Experiment

The optimal adsorbent dosage was determined based on our preliminary investigations. Various amounts of adsorbents, specifically 0.01 g, 0.02 g, 0.04 g, 0.06 g, 0.08 g, and 0.1 g, were employed to evaluate the adsorption performance. Among these, the adsorbent dosage of 0.01 g exhibited the highest adsorption capacity. Therefore, in this study, for the adsorption capacity measurement of LR-EPI hydrogels for Pb^2+^, Cu^2+^, and Cr^3+^, 0.01 g of freeze-dried LR-EPI hydrogel samples were added to a sealed colorimetric tube containing 25 mL of heavy metal solutions. The adsorption experiment was conducted at different pH values ranging from 1.0 to 5.0. Nitric acid and sodium hydroxide solutions were used to adjust the pH accordingly. In the adsorption kinetics study, the contact time between the hydrogel samples and heavy metal solutions varied from 10 to 120 min. This allowed for the examination of the adsorption process over different time intervals. In the adsorption isotherm experiment, the concentrations of heavy metal ions in the solutions ranged from 100 to 1000 mg/L. After the adsorption process, the remaining concentrations of heavy metal ions were measured using an atomic absorption spectrophotometer (PerkinElmer, PinAAcle 900 T, Waltham, MA, USA). Specifically, the concentrations of mixed metal ions in both the selective adsorption experiment and real water matrices were analyzed using an inductively coupled plasma mass spectrometer (ICP-MS, Agilent, Santa Clara, CA, USA). All experimental data presented in this study represent the average values obtained from three independent determinations.

The adsorption capacity of the hydrogels for Pb^2+^, Cu^2+^, and Cr^3+^ at a specific time (*q_t_*, mg/g) and equilibrium (*q_e_*, mg/g) was determined by the following equations [72]:

(7) $$q_{e}=\frac{(C_{0}{-C}_{e})V}{W}$$

(8) $$q_{t}=\frac{(C_{0}{-C}_{t})V}{W}$$

where *q_e_* (mg/g) is the equilibrium adsorption capacity, *q_t_* (mg/g) is the adsorption capacity at a specific time *t*, *C*_0_ (mg/L) is the initial concentration, *C_e_* (mg/L) is the equilibrium concentration, *C_t_* (mg/L) is the concentration of the heavy metal solution at time *t* (h), *V* (mL) is the volume of the heavy metal ion solution, and *W* (mg) is the amount of freeze-dried hydrogel.

### 4.7. The Reusability of Hydrogels

A freeze-dried LR-EPI-8 hydrogel weighing 0.01 g was immersed in a 25 mL aqueous solution containing Pb^2+^ or Cu^2+^ ions at pH 5 under static conditions (25 °C). The adsorption process was assessed, and the reusability of the hydrogel was determined. Following the adsorption step, the hydrogels were separated from the Pb^2+^ or Cu^2+^ solution and subsequently subjected to desorption and regeneration procedures. To desorb the Pb^2+^ or Cu^2+^ ions from the hydrogel, a 0.1 mol/L HCl aqueous solution was employed and allowed to interact for a duration of 3 h. After desorption, the hydrogels were thoroughly washed with distilled water to facilitate utilization for subsequent adsorption cycles. This adsorption–desorption cycle was repeated five times to evaluate the reusability and durability of the hydrogels.

## Figures and Tables

**Table 1:** Adsorption of heavy metal ions by different adsorbents.

Adsorbent	*q_m_* (mg/g)			References
	Pb^2+^	Cr^3+^	Cu^2+^	
Licorice/epichlorohydrin	591.75	583.5	134.25	Present study
Chitosan/calcium alginate/bentonite	434.89	-	115.30	[62]
Chitosan/poly(N-isopropylacrylamide)	172.0	-	115.1	[63]
Chitosan/polyvinyl alcohol	-	-	62.1	[64]
Spirodela polyrhiza/epichlorohydrin	51.75	-	-	[65]
N,N-dimethylformamide				
Carboxymethyl cellulose/attapulgite/acrylic acid-co-acrylamide	-	74.8	-	[66]
Cellulose/epichlorohydrin/graphene oxide	-	-	94.34	[9]
Tea residue/acrylic acid	253.16	206.1	-	[67]
Carboxymethyl cellulose/epichlorohydrin/polyacrylamide	-	-	75.93	[68]
Silica/poly(N-isopropylacrylamide)				
N,N′-methylenebis-acrylamide	-	243.9	-	[69]

## Data Availability

The data presented in this study are available upon request from the first author (X.Y.) and corresponding author (H.W.).

## Supplementary Materials

The following supporting information can be downloaded at: https://www.mdpi.com/article/10.3390/gels9070559/s1, Figure S1: The photos of Liquorice. (a) raw Liquorice; (b) LR-NaOH; (c) LR- NaOH powder. Figure S2. Photographs of (a) LR-EPI-5; (b) LR-EPI-6; (c) LR-EPI-8; (d) lyophilized LR-EPI-5; (e) lyophilized LR-EPI-6; (f) lyophilized LR-EPI-8. Figure S3. Correlation between Zeta potential and pH. Figure S4. Thermogravimetric analysis of LR-EPI -8 before and after adsorption (Cr^3+^). Figure S5. The Intra-particle Diffusion model of Cu^2+^ adsorption on hydrogel adsorbents. Figure S6. The Intra-particle Diffusion model of Pb^2+^ adsorption on hydrogel adsorbents. Figure S7. The Intra-particle Diffusion model of Cr^3+^ adsorption on hydrogel adsorbents. Figure S8. Langmuir and Freundlich isotherm non-liner models of LR-EPI. Table S1. Kinetic parameters for the adsorption of heavy metal ions. Table S2. Parameters of the Intra-particle Diffusion model for Cu^2+^ adsorption on hydrogel adsorbents. Table S3. Parameters of the Intra-particle Diffusion model for Pb^2+^ adsorption on hydrogel adsorbents. Table S4. Parameters of the Intra-particle Diffusion model for Cr^3+^ adsorption on hydrogel adsorbents. Table S5. Adsorption isotherms of heavy metal ions on LR/EPI hydrogel. Table S6. Thermodynamic parameters of LR-EPI-8 for Pb(II) and Cu(II). Table S7. The parameters of non-liner isotherms models of LR-EPIs.
