# Supplementary material for: Efficient Removal of Heavy Metals from Aqueous Solution Using Licorice Residue-Based Hydrogel Adsorbent
Source: Gels. 2023 Jul 9;9(7):559. doi: 10.3390/gels9070559 (PMC10379308; doi:10.3390/gels9070559)
Supplement: Supplementary file 1 [file gels-09-00559-s001.zip › gels-2455928-supplementary.pdf]

Efficient removal of heavy metals from aqueous solution using Licorice residue-based hydrogel adsorbent

Xiaochun Yin [a,b*], Ting Ke[b], Hai Zhu[b], Pei Xu[a] and Huiyao Wang[a*]

[a] Department of Civil Engineering, New Mexico State University; Las Cruces, NM 88003, USA
[b] School of Public Health, Gansu University of Chinese; Lanzhou, Gansu 730000, China

Contents:

Figure S1. Photos of licorice: (a) raw licorice; (b) LR-NaOH; (c) LR- NaOH powder.

Figure S2. Photographs of (a) LR-EPI-5; (b) LR-EPI-6; (c) LR-EPI-8; (d) lyophilized LR-EPI-5; (e) lyophilized LR-EPI-6; (f) lyophilized LR-EPI-8.

Figure S3. Correlation between zeta potential and pH.

Figure S4. Thermogravimetric analysis of LR-EPI-8 before and after adsorption (Cr^{3+}).

Figure S5. The intra-particle diffusion model of Cu^{2+} adsorption on hydrogel adsorbents.

Figure S6. The intra-particle diffusion model of Pb^{2+} adsorption on hydrogel adsorbents.

Figure S7. The intra-particle diffusion model of Cr^{3+} adsorption on hydrogel adsorbents.

Figure S8. Langmuir and Freundlich isotherm non-liner models of LR-EPI.

Table S1. Kinetic parameters for the adsorption of heavy metal ions.

Table S2. Parameters of the intra-particle diffusion model for Cu^{2+} adsorption on hydrogel adsorbents.

Table S3. Parameters of the intra-particle diffusion model for Pb^{2+} adsorption on hydrogel adsorbents.

Table S4. Parameters of the intra-particle diffusion model for Cr^{3+} adsorption on hydrogel adsorbents.

Table S5. Adsorption isotherms of heavy metal ions on LR/EPI hydrogels.

Table S6. Thermodynamic parameters of LR-EPI-8 for Pb(II) and Cu(II).

Table S7. The parameters of non-liner isotherms models of LR-EPIs.

* Corresponding authors:
Email address: Huiyao@nmsu.edu (Huiyao Wang); lzyxc@nmsu.edu (Xiaochun Yin)

1.1 Morphology of **Licorice and pretreated Licorice residues**

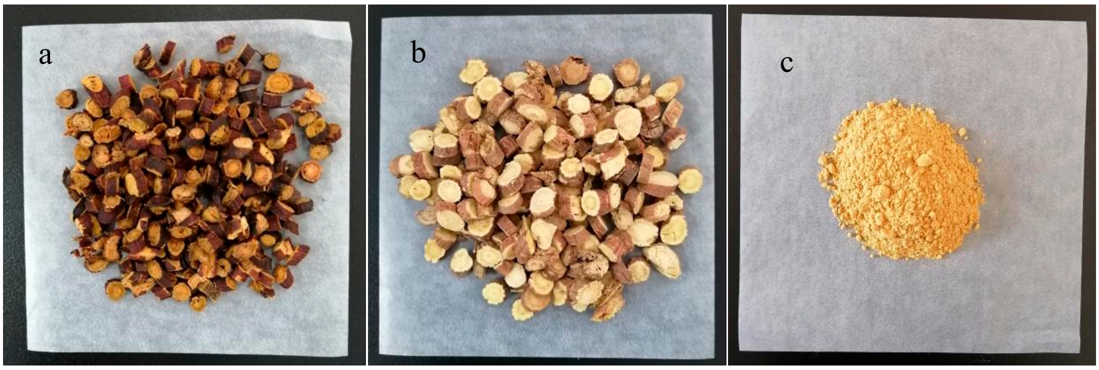

Figure S1. Photos of licorice: (a) raw licorice; (b) LR-NaOH; (c) LR- NaOH powder.

1.2 Morphology of LR-EPI hydrogels

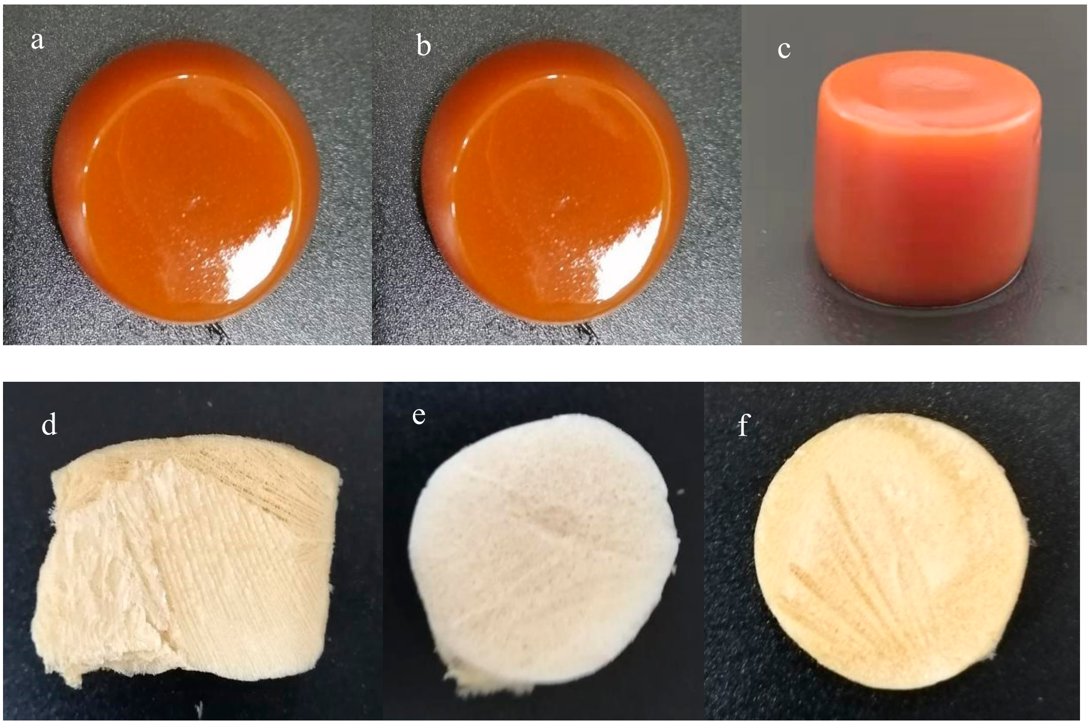

Figure S2. Photographs of (a) LR-EPI-5; (b) LR-EPI-6; (c) LR-EPI-8; (d) lyophilized LR-EPI-5; (e) lyophilized LR-EPI-6; (f) lyophilized LR-EPI-8.

1.3 Zeta potential analysis

The zeta potential of hydrogels in different pH solutions was tested (Figure S3). The results show that within the measured range (2-5), the zeta potential was always negative, indicating that the obtained hydrogels always carry a negative charge in different pH solutions. It was also found that the zeta potential decreased gradually with the gradual increase in the pH. This result indicates that the surface adsorption active sites of the hydrogels increased from a strong acid solution to a neutral solution.

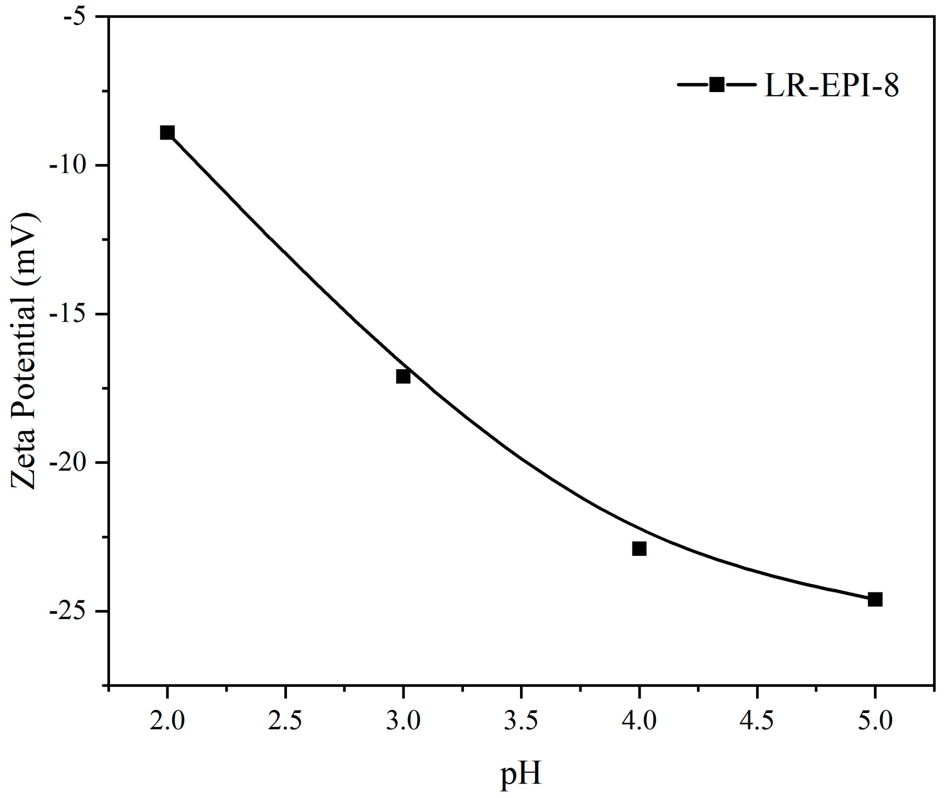

Figure S3. Correlation between zeta potential and pH.

1.4 Thermogravimetric Analysis

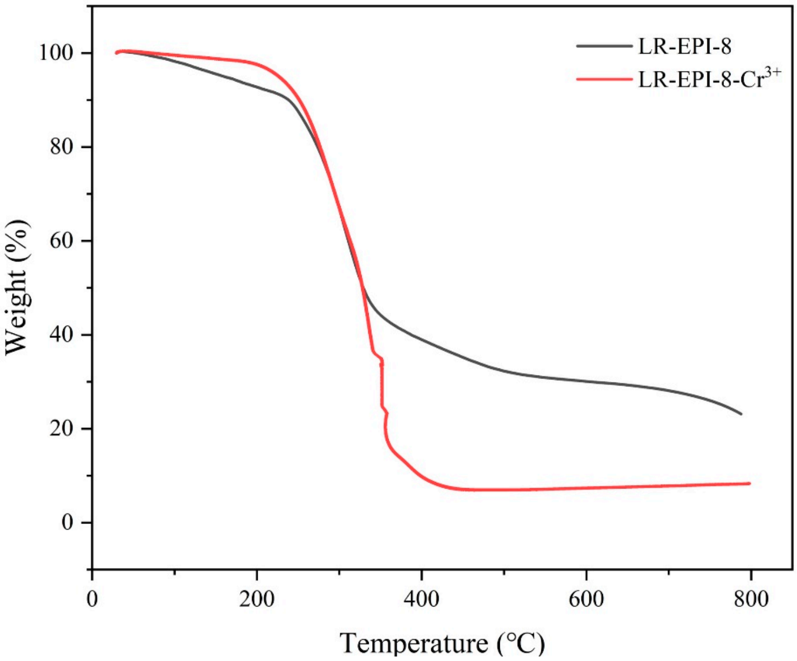

Figure S4. Thermogravimetric analysis of LR-EPI-8 before and after adsorption (Cr^{3+}).

1.4 Adsorption kinetic parameters

Table S1. Kinetic parameters for the adsorption of heavy metal ions.

	Pseudo-first-order				Pseudo-second-order			
	$k_1(min^{-1})$	q_{e1}(mg/g)	R^2	*RMSE*	k_2 (g /mg-min)	q_{e2}*(mg/g)*	R^2	*RMSE*
Pb(II)	0.0371	278.53	0.9904	0.0242	0.0002	617.28	0.9999	0.0006
Cr(III)	0.0309	291.81	0.9174	0.1422	0.0001	529.10	0.9615	0.0115
Cu(II)	0.0261	28.15	0.8300	0.1551	0.0025	134.05	0.9990	0.0080

1.5 Intra-particle diffusion model

The intra-particle diffusion model is useful in studying the diffusion process that happens in the surface layer during adsorption kinetics. By using this model, we can better understand how substances diffuse in the surface layer and how this affects other materials. We have extensively researched this model to gain insights into the diffusion mechanism during adsorption. This model considers the availability of adsorption sites and

mass transfer processes, which are the main factors that affect adsorption kinetics. The equation for the intra-particle diffusion model is as follows (1):

$$Q_t = k_i t^{1/2} + C \qquad (1)$$

where *Qt* (mg/g) is the adsorption capacity at a specific time; *ki* (mg/g·min$^{0.5}$) is the intra-particle diffusion constant associated with the absorption rate; and C (mg/g) is the intercept.

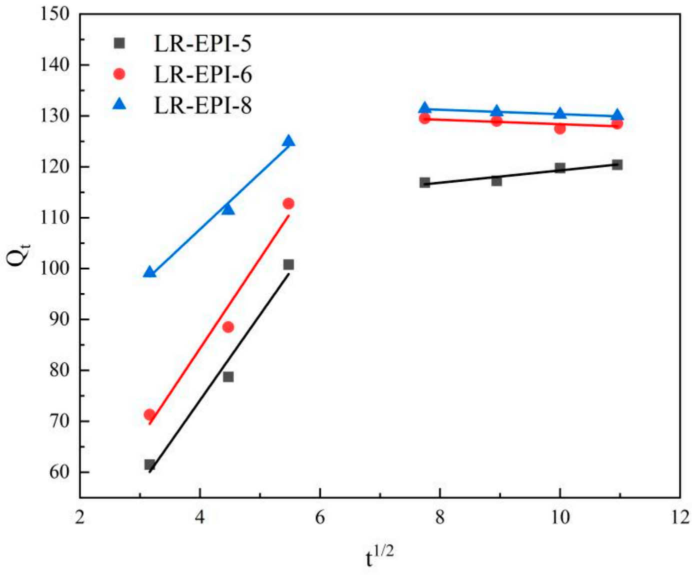

Figure S5. The intra-particle diffusion model of Cu^{2+} adsorption on hydrogel adsorbents**.**

Table S2. Parameters of the intra-particle diffusion model for Cu^{2+} adsorption on hydrogel adsorbents.

	k_{i1} (mg/g·min$^{0.5}$)	C_1*(mg/g)*	R^2	k_{i2}(mg/g·min$^{0.5}$)	C_2*(mg/g)*	R^2
LR-EPI-5	16.79	6.97	0.9575	1.21	107.19	0.8530
LR-EPI-6	17.67	13.62	0.9402	-0.43	132.72	0.2419
LR-EPI-8	11.04	63.56	0.9785	-0.44	134.70	0.9741

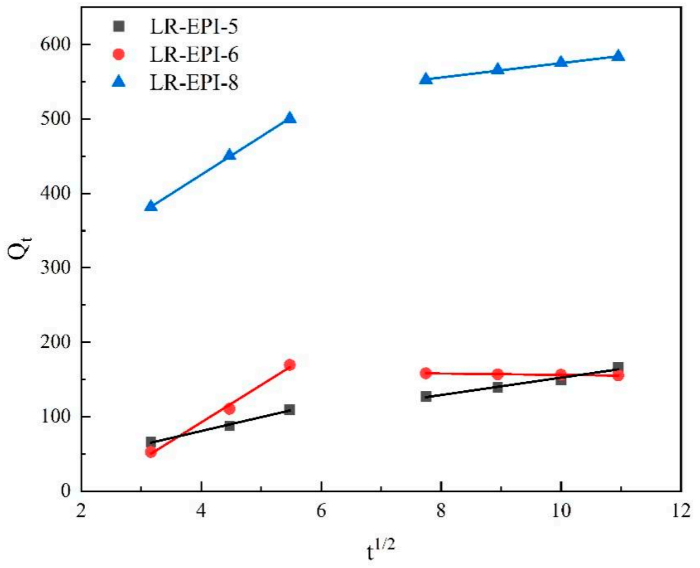

Figure S6. The intra-particle diffusion model of Pb^{2+} adsorption on hydrogel adsorbents**.**

Table S3. Parameters of the intra-particle diffusion model for Pb^{2+} adsorption on hydrogel adsorbents.

	k_{i1} (mg/g·min$^{0.5}$)	C_1(mg/g)	R^2	k_{i2} (mg/g·min$^{0.5}$)	C_2(mg/g)	R^2
LR-EPI-5	18.68	5.90	0.9889	11.68	35.62	0.9590
LR-EPI-6	50.23	-108.72	0.9867	-0.92	165.29	0.9576
LR-EPI-8	51.40	219.36	0.9994	9.66	478.38	0.9949

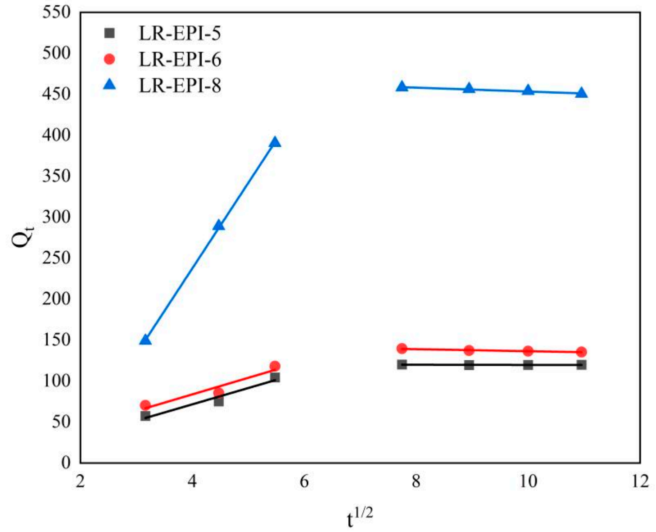

Figure S7. The intra-particle diffusion model of Cr^{3+} adsorption on hydrogel adsorbents**.**

Table S4. Parameters of the intra-particle diffusion model for Cr^{3+} adsorption on hydrogel adsorbents.

	k_{i1}(mg/g·min$^{0.5}$)	C_1(mg/g)	R^2	k_{i2}(mg/g·min$^{0.5}$)	C_2(mg/g)	R^2
LR-EPI-5	19.93	-8.06	0.9077	-0.11	120.88	0.8826
LR-EPI-6	20.24	2.84	0.8408	-1.23	148.82	0.9281
LR-EPI-8	104.32	-179.71	0.9995	-2.38	477.16	0.9517

1.6 Adsorption isotherm parameters

Table S5. Adsorption isotherms of heavy metal ions on LR/EPI hydrogels.

	Langmuir					Freundlich			
	q_m(mg/g)	K_L(L/mg)	R_L	R^2	RMSE	n	K_F($mg^{1-1/n}$ $L^{1/n}$ g^{-1})	R^2	RMSE
Pb(II)	632.91	0.0129	0.2059	0.9856	0.0586	3.26	79.61	0.6669	0.1595
Cr(III)	2504.40	0.0004	0.8010	0.2457	0.2266	1.05	1.03	0.9393	0.1901
Cu(II)	151.29	0.0076	0.1582	0.9937	0.3912	3.11	15.53	0.9180	0.1111

Thermodynamic parameters were calculated using:

$$ln\, k_L = \frac{\Delta S^\circ}{R} - \frac{\Delta H^\circ}{RT} \qquad (3)$$

where R is the universal gas constant (8.314×10^{-3} kJ/mol·K), T is the temperature (K), and k_L is the Langmuir adsorption equilibrium constant (L/mg).

Table S6. Thermodynamic parameters of LR-EPI-8 for Pb(II) and Cu(II).

Gel	Metal ions	lnk_L	ΔG°(kJ/mol)	ΔS°(J/mol·K)	ΔH°(kJ/mol)
LR-EPI-8	Pb(II)	4.35	-10.78	41.27	1.52
	Cu(II)	3.64	-9.02	34.33	1.21

1.7 non-linear adsorption isotherm models

The non-linear isotherm models of Langmuir and Freundlich are shown in Figure S8 and Table S7. The adsorption process of the three heavy metals does not fit the Langmuir and Freundlich models because the R^2 values are all smaller than 0.95, and the RMSE is too large. So, the non-linear isotherm model's fit was not

used to describe the adsorption.

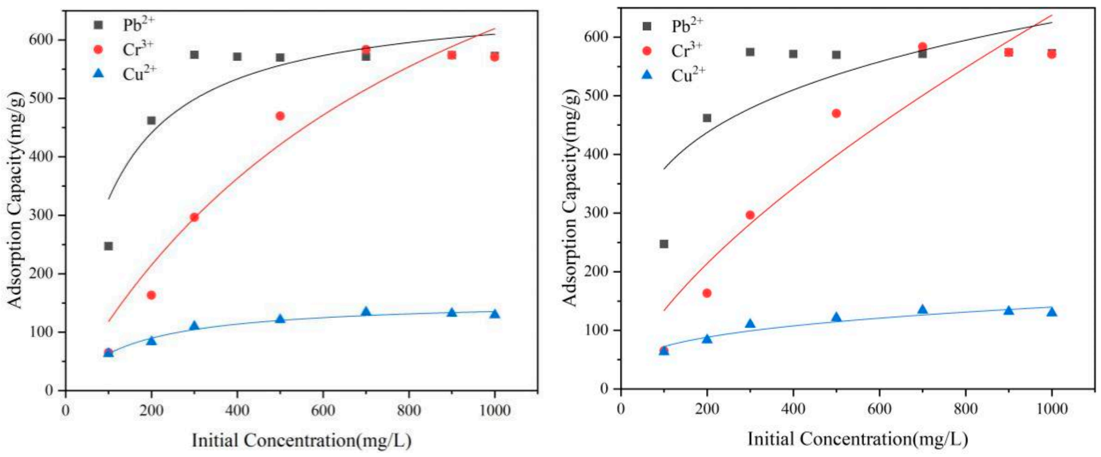

Figure S8. Langmuir and Freundlich isotherm non-liner models of LR-EPI.

Table S7. The parameters of non-liner isotherm models of LR-EPIs.

	Langmuir					Freundlich			
	$q_m(mg/g)$	$K_L(L/mg)$	R_L	R^2	$RMSE$	n	$K_F\ (mg^{1-1/n}\ L^{1/n}\ g^{-1})$	R^2	$RMSE$
Pb(II)	574.5	0.0016	0.8621	0.9856	0.0586	4.38	125.18	0.6669	0.1595
Cr(III)	583.5	0.0070	0.9346	0.2457	0.2266	3.03	97.85	0.9393	0.1901
Cu(II)	134.25	0.0069	0.5917	0.9972	0.1185	2.63	16.27	0.9622	0.0510
